# A lipophilic fluorescent LipidGreen1-based quantification method for high-throughput screening analysis of intracellular poly-3-hydroxybutyrate

**DOI:** 10.1186/s13568-015-0131-6

**Published:** 2015-08-09

**Authors:** Ji Eun Choi, Hye Young Na, Taek Ho Yang, Sung-Keun Rhee, Jae Kwang Song

**Affiliations:** Research Center for Bio-based Chemistry, Korea Research Institute of Chemical Technology (KRICT), 141 Gajeong-ro, Yuseong-gu, Daejeon, 305-600 Republic of Korea; Department of Microbiology, Chungbuk National University, Chungdae-ro 1, Seowon-gu, Cheongju, Chungbuk 362-763 Republic of Korea; GenoFocus, 65 Techno 1-ro, Yusung-gu, Daejeon, 305-509 Republic of Korea

**Keywords:** LipidGreen1, Poly-3-hydroxybutyrate, High-throughput screening, Quantitative measurement, Spectrofluorometry, Microtiter plate assay

## Abstract

Poly-3-hydroxybutyrate (PHB), the most abundant type of polyhydroxyalkanoates (PHA) is synthesized inside a variety of microorganisms as a primary candidate for industrial PHB production. Lipophilic dyes such as Nile red and BODIPY have been used to quantify intracellular PHB, but their uses have often been limited in terms of sensitivity and accuracy. In this study, a newly developed lipophilic fluorescent dye LipidGreen1 was used to quantify intracellular PHB. LipidGreen1 stained viable colonies by adding the dye into the medium which enabled the effective selection of PHB-positive cells. Furthermore, the fluorescence intensity of LipidGreen1 maintained its fluorescence intensity much longer than that of Nile red. The fluorescence intensities of intracellular PHB stained by LipidGreen1 accurately agreed with PHB contents measured by gas chromatography. In addition, internalization of LipidGreen1 in *Escherichia coli* cell was not necessary to obtain quantitative measurements. PHB-synthase mutants were differentiated by fluorescence intensities with a good correlation to increased levels of PHB production. These results show that LipidGreen1 is sensitive and accurate in high-throughput screening of newly isolated and genetically modified bacteria with enhanced PHB production.

**Electronic supplementary material:**

The online version of this article (doi:10.1186/s13568-015-0131-6) contains supplementary material, which is available to authorized users.

## Introduction

Polyhydroxyalkanoate (PHA) is a linear polyester which accumulates in various gram-positive and gram-negative bacteria as an intracellular granular material for carbon and energy storage from renewable resources (Snell and Peoples 2009). Bacterial PHA shows a good promise as a biodegradable and biocompatible plastic for packaging and medical applications (Reddy et al. 2013). Therefore, it is considered as a functional substitute for petroleum-based plastics due to thermoplastic and elastomeric properties of its copolymers (Balaji et al. 2013). Poly-3-hydroxybutyrate (PHB), the most abundant type of PHA, can be produced from a variety of sugars. For example, *Cupriavidus necator* H16 (formerly, *Ralstonia eutropha* H16) synthesizes PHB from acetyl-CoA through three enzyme reactions (Peoples and Sinskey 1989). PHB production from *Cupriavidus necator* H16 has been studied in many fields, including the application of non-edible carbon sources into PHB production such as food wastes (Hafuka et al. 2010) and Jatropha oil (Ng et al. 2010). Furthermore, PHB production has been well studied with functional genes of *Cupriavidus necator* H16 (Budde et al. 2010; Kahar et al. 2004). Recently, many strategies including culture medium manipulations (Khanna and Srivastava 2005; Nath et al. 2008) and genetic modifications (Madison and Huisman 1999; Lim et al. 2002) have been developed to increase PHB production. PHB synthesis in recombinant bacteria is considered to be economically beneficial due to its fast growth rate and high accumulation of PHB up to 90% of its dry cell weight and thus, has been thoroughly investigated in genetic engineering and culture optimization studies to enhance PHB-productivity (Kim et al. 1992; Slater et al. 1988).

It is necessary to develop better enzymes relevant to PHB biosynthesis and identify high-yield production strains. Thus, a simple and reliable high-throughput method, having the advantage of real time monitoring of cell growth and PHB contents, is needed. Although chromatographic analysis provides the most accurate details relative to PHB quantification and monomer composition, it involves the complex and time-consuming steps such as the extraction and derivatization of PHB. Therefore, it is not suitable for high-throughput measurements of a large number of samples. Nowadays, lipophilic fluorescent dyes such as Nile Red (a benzophenoxazone dye), BODIPY (a boron-dipyrromethene dye) (Cirulis et al. 2012; Tyo et al. 2006; Pinzon et al. 2011) are generally used as a rapid and high-throughput detection method. Nile red has been used to measure PHB contents inside microbial cells with a micro-fluorospectrometer (Schlebusch and Forchhammer 2010; Zuriani et al. 2013) and fluorescence activated cell sorter (FACS) (Kacmar et al. 2005; Tyo et al. 2006). However, the use of Nile red has low sensitivity and poor reliability, when it is used with viable cells growing in a liquid culture medium and entrained in a FACS system (Lee et al. 2013).

LipidGreen1 is a new small fluorescence probe with an indolin-3-one skeleton, which successfully stained lipid droplets in 3T3-L1 and HepG2 cells and fat deposits in zebrafish (Chun et al. 2013; Lee et al. 2011). LipidGreen1 could be used to detecting bacterial polyesters including PHB. In this study, we suggested that LipidGreen1 is a powerful tool for rapid and accurate selection of enhanced PHB-producing bacteria with micro-fluorospectrometer. Furthermore, the PHB contents of PHA synthase mutant library could be measured using the high-throughput LipidGreen1 staining method.

## Materials and methods

### Plasmids, bacteria and chemicals

The plasmid pPhaCAB consists of a pBluescript II SK+ backbone (Stratagene, USA) and the PHB biosynthetic gene cluster encoding three genes for type I PHA synthase (*phaC*), ketothiolase (*phaA*), and acetoacetyl-CoA reductase (*phaB*) from *Cupriavidus necator* H16 (Yang et al. 2010). *Escherichia coli* XL1-Blue (Stratagene) was transformed with pPhaCAB for expression of the PHA biosynthesis genes. LipidGreen1 was provided by Korea Chemical Bank (KRICT, South Korea; Additional file 1: Fig. S1). LipidGreen1and Nile red stock solutions were prepared by dissolving the dyes in dimethylsulfoxide (DMSO) to a final concentration of 1 mg/mL. PHB powder was purchased from Sigma-Aldrich (USA). Ten milligrams PHB powder was suspended in 1 mL water using ultrasonic homogenizer (Sonics and Materials, USA) for 1 min on 20% amplitude.

### Culture conditions

Recombinant *E. coli* XL1-Blue transformed with phaCAB, PHB-producing cell, was grown at 37°C in Luria–Bertani (LB) medium containing 10 g/L tryptone, 5 g/L yeast extract, 5 g/L NaCl, and 50 μg/mL ampicillin. After 20 h cultivation in 2 mL of LB broth, the PHB-producing cells were inoculated into LB medium supplemented with 20 g/L glucose and cultured on an incubator at 37°C for 20 h with shaking (200 rpm). For cell viability analysis, the PHB-producing cells were cultivated in 100 mL LB medium with 20 g/L glucose and LipidGreen1 (0, 0.8, and 2 µg/mL). The cultures were collected every 2 or 3 h and then optical densities at 600 nm were measured (Shimadzu, Japan).

### Observation of bacterial PHB on an agar plate

The PHB-producing cells were spread on the agar plate containing LipidGreen1 at a final concentration of 25 µg/mL and cultured for 20 h at 37°C. Accumulation of intracellular PHB was viewed under ultraviolet light (302 nm). *E. coli* XL1-Blue, which contains only the pBluescript II SK+ vector (Agilent Technologies, USA), was prepared as a negative control. Subsequently, the PHB-producing and PHB-non-producing cells were scraped from the surface of the agar plates and suspended in 100 µL of phosphate-buffered saline (PBS, pH 7.2, 20 mM). Ten microliters of the suspensions were placed on slide glass and used for microscopic observation by fluorescence microscope (Nikon, Japan) with a green fluorescence filter (Green Excitation 460–500 nm, Emission 510–560 mm).

### Measurement of the fluorescence intensity

The PHB-producing cells were cultivated in 500 mL LB medium containing 20 g/L glucose. The cells were harvested by centrifugation (3,200×*g*, 4°C for 10 min) and resuspended in PBS to yield an optical density at 600 nm of 2.0. LipidGreen1 was added to the 1 mL cell suspensions, followed by further incubation for 0.5 and 2 h in the dark. The final concentration of LipidGreen1 at 2 µg/mL was used in further experiment. One hundred microliters of the suspensions were immediately transferred into a 96 well black microplate, and the fluorescence intensity was measured within 10 min with a micro-fluorospectrometer (TECAN, Switzerland) at an excitation wavelength of 450 nm and emission wavelength of 510 nm. To verify the relation between the PHB accumulation contents and fluorescence intensity, 30 mL of the culture solutions were collected at 2 h intervals during cell growth and stored at −70°C deep freezer for fluorescence and GC analysis. In addition, the aqueous PHB suspension was serially diluted in water and then incubated with LipidGreen1 for 30 min in black microtubes followed by measurement of fluorescence intensities. Furthermore, Nile red was added to the 1 mL cell suspensions to give a final concentration of 2 µg/mL, and then fluorescence intensities were measured at 540 and 570 nm for the excitation and emission wavelengths, respectively.

### Comparison of fluorescence intensity between intact and lysed cells

The PHB-producing cells grown in the 50 mL medium were collected by centrifugation (4°C at 3,200×*g* for 10 min) to an optical density at 600 of 4.0. Half of the cell suspension in PBS buffer was disrupted with the ultrasonic homogenizer, while the remaining suspension was left on ice as intact cells. One milliliter each of disrupted and intact cell suspension was moved into a black microtube followed by the addition of LipidGreen1. The mixtures were incubated for 0.5 and 2 h and immediately transferred into a black 96-well microplate to measure the fluorescence intensity.

### PHB quantification by gas chromatography (GC)

The PHB polymer content was determined by GC analysis as previously described (Yang et al. 2010). Briefly, PHB-producing cells were washed twice with PBS buffer and dried at 65°C in an oven with a final dry pellet weight of 0.03 g. The dry matter was subjected to methanolysis in the presence of 1 mL PHA solution containing 0.8% (wt/vol) Benzoic acid, 3% (vol/vol) sulfuric acid, 97% (vol/vol) methanol and 2 mL of chloroform. Following 6 h of incubation at 100°C, the polymer solutions dissolved in chloroform were precipitated and separated with chilled deionized water. The PHB contents were analyzed by GC (6890N GC system, Agilent Technologies) equipped with a fused silica capillary column (SPBTM-5, 30 m × 0.32 mm ID, 0.25 µm film; Supelco, USA) using benzoic acid as an internal standard.

### Construction and screening of a *phaC* mutant library

Random mutagenesis was performed by error-prone PCR with GeneMorph II Random mutagenesis kit (Stratagene) following the manufacturer’s instructions. Briefly, to introduce random mutations into the *phaC* gene, forward and reverse primers (ReCMutF; 5′-GATCCCCCGGGCAAGTACC-3, ReCMutR; 5′-GGGAACCTGCAGGCCTGC-3′) were designed based on the nucleotide sequences outside of the structural gene. Each PCR contained 30 ng of the pPhaCAB plasmid as the initial template, 250 ng of each primer, 200 μM of each dNTP, and 5 U of Taq DNA polymerase in Taq DNA polymerase reaction buffer. The PCR started with a denaturation step at 95°C for 30 s, followed by 25 cycles of amplification (30 s at 95°C, 30 s at 55°C, and 3 min at 72°C), and a final extension step at 72°C for 10 min. The PCR products purified with the QIAquick PCR Purification Kit (Qiagen, USA) were digested with SmaI and SbfI and subjected to preparative electrophoresis in a 0.8% agarose gel. The approximately 1.8-kb PCR fragments were ligated into the same restriction sites of the pPhaCAB vector, and then the ligates were transformed into *E. coli* XL1-Blue. Each mutant clone was grown in a deep-well microplate containing 800 µL of the LB medium containing 20 g/L glucose for 20 h, of which 100 µL were transferred to a black microplate to measure its fluorescence intensity by adding LipidGreen1. Determination of the cellular PHB content by GC was done with selected clones that showed relatively higher or lower fluorescence intensities than that of the wild type.

## Results

### LipidGreen1 staining of PHB-producing bacterial cells on agar plates

**Fig. 1:**
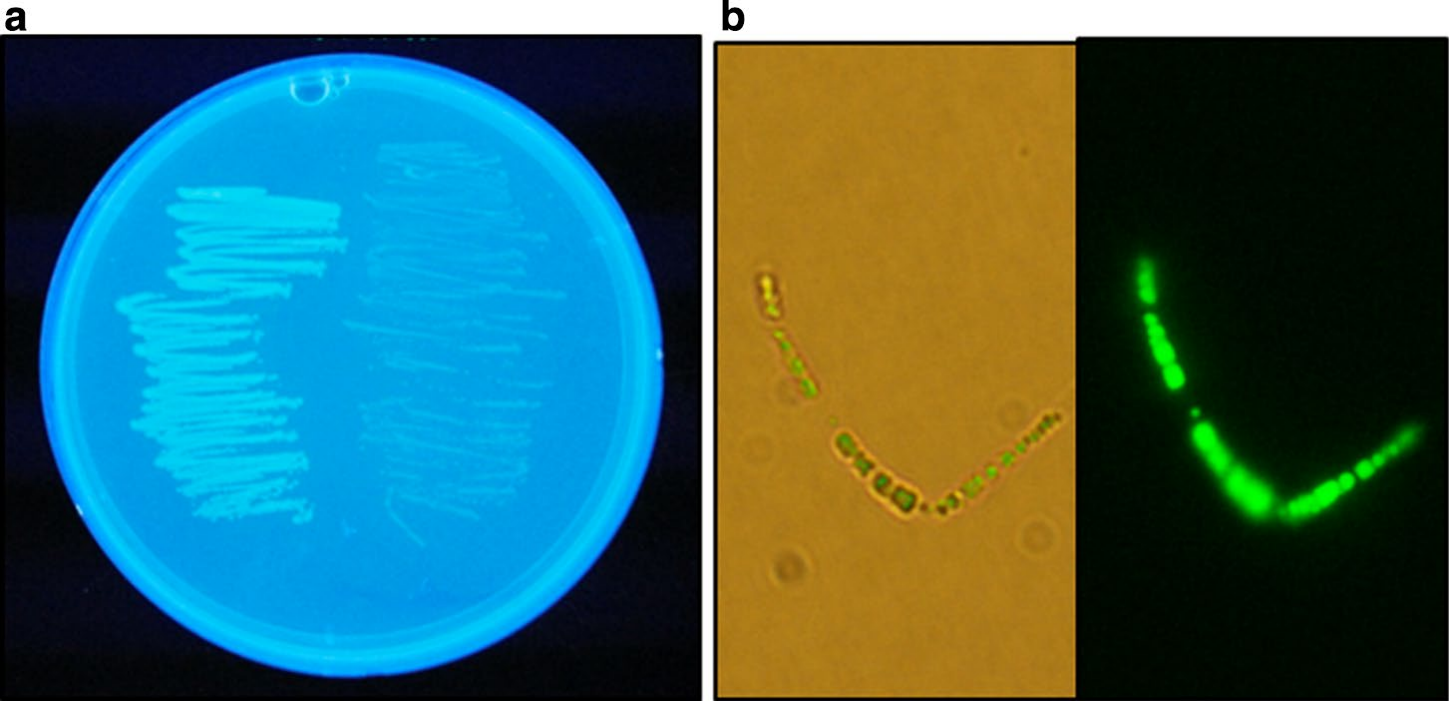
Fluorescence of PHB-producing cells grown on a LipidGreen-containing agar plate. **a** Fluorescence staining of PHB-producing and PHB-nonproducing cells on an agar plate. *E. coli* harboring pPhaCAB (*left side* of the plate) had bright fluorescence under UV light (302 nm) in contrast to *E. coli* containing pBluescript II SK+ vector (*right side* of the plate) exhibiting a much weaker fluorescence than above. **b** Microscopic observation of *E. coli* stained by LipidGreen1 on agar plates. The cell suspension in PBS buffer was placed on a glass slide to obtain optic (*left*) and fluorescent (*right*) images. The images were viewed under the fluorescence microscope (×1,000 amplification) with a *green* fluorescence filter.

LipidGreen1, a novel fluorescent dye previously used to stain neutral lipids and fat deposits in eukaryotic cells and tissues (Lee et al. 2011), has a unique chemical structure consisting of an indoline-3-one skeleton. The core skeleton of LipidGreen1 is markedly different from a benzophenoxazine skeleton of the representative lipophilic fluorescent dye, Nile red (Additional file 1: Fig. S1). We examined LipidGreen1 staining for PHB accumulated inside bacterial cells. When the *E. coli* cells harboring pPhaCAB were grown for 20 h on agar plates containing 25 µg/mL LipidGreen1, they exhibited a far stronger fluorescence than the *E. coli* cells harboring pBluescript II SK(+) vector (Fig. 1a). The *E. coli* cells stained with LipidGreen1 on agar plates were collected, suspended in PBS buffer (pH 7.2), and observed with fluorescence microscopy (Fig. 1b). The strong fluorescence of the PHB-producing *E. coli* cells also indicated that the LipidGreen1 was capable of detecting the PHB accumulation of *E. coli* cell grown on agar plates and that its fluorescent signal was maintained for quite a long time.

### LipidGreen1 staining of PHB-producing bacterial cells in liquid medium

**Fig. 2:**
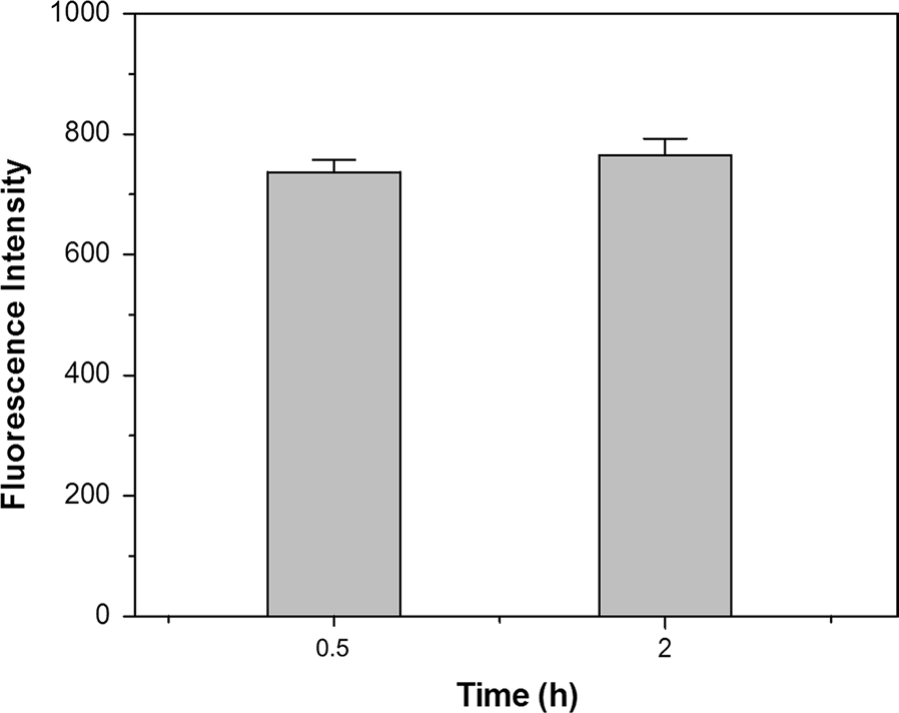
Comparison of fluorescence intensity for LipidGreen1 at different time points. A PHB-producing cell suspension at OD_600_ 2.0 was stained with LipidGreen1 at a final concentration of 2.0 µg/ml. *Gray bars* are fluorescence intensities for incubation times of 0.5 and 2 h after adding LipidGreen1, respectively. The data shown are the means with standard deviation (*error bars*) from three independent experiments.

**Fig. 3:**
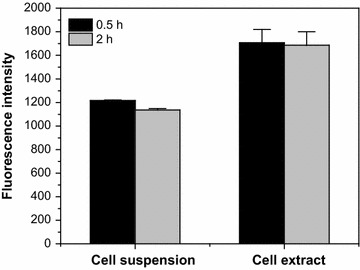
Differences in fluorescence intensity between cell suspensions and cell extracts. Fully grown PHB-producing cells were resuspended into PBS to an OD_600_ of 4.0. Fluorescence intensity was estimated using non-treated cells and lysed cells by ultrasonication after prolonged incubation for 0.5 and 2 h.

After PHB-producing cells were grown in the medium, LipidGreen1 to a final concentration of 2 µg/mL was added to the cell suspension and then incubated for 0.5 and 2 h. The fluorescence intensity of LipidGreen1 was 740 in PHB-producing cells (Fig. 2), whereas that of the *E. coli* cells containing none of PHB was 248 (data not shown). In particular, the fluorescence intensity of LipidGreen1 was stably maintained for 2 h. On the other hand, the fluorescence intensity of Nile red as a representative fluorescent lipophilic dye was significantly decreased only after 1 h incubation with the PHB-producing cells (Additional file 1: Fig. S2). We also found out that LipidGreen1 was not harmful to the cell growth (Additional file 1: Fig. S3-A). When the PHB-producing *E. coli* was grown in the LB medium containing 20 g/L glucose and LipidGreen1 up to the final concentration of 2 µg/mL, the cell growth in the presence of LipidGreen1 (0.8 and 2.0 µg/mL) was similar to the cell growth in the absence of LipidGreen1. Also, PHB-producing cells showed similar growth between Nile red containing (0, 0.8, 2 µg/mL) and non-containing PHB-producing medium (Additional file 1: Fig. S3-B). Differences in the fluorescence intensity between membrane-disrupted and non-disrupted cells were measured to assess the LipidGreen permeability to cell membrane. Ultrasonication to *E. coli* cells caused an increase in fluorescence intensity (Fig. 3). However, regardless of whether the cells were disrupted or not, the prolonged incubation for 0.5 and 2 h after adding LipidGreen1 did not influence the fluorescence intensity.

### Quantitative correlation between fluorescence intensity and PHB contents

**Fig. 4:**
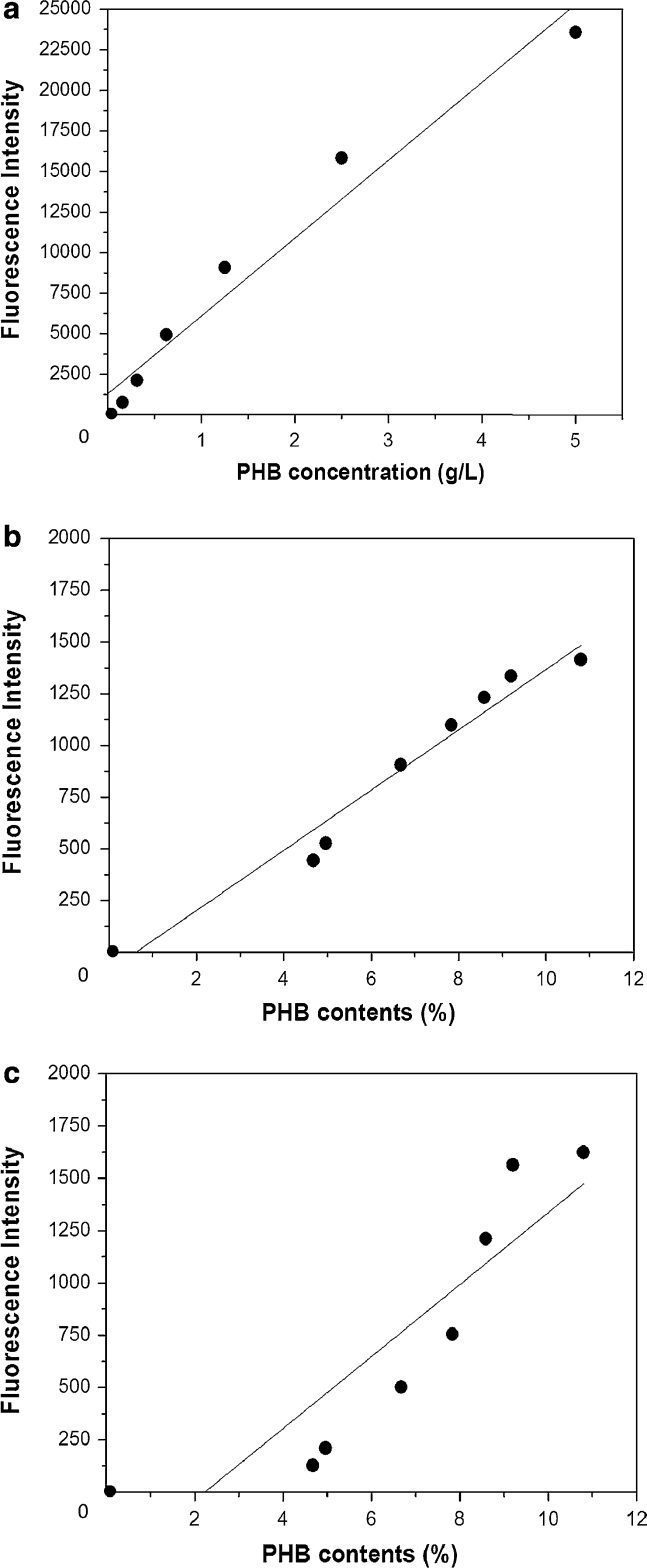
Fluorescence intensities of PHB powder and intracellular PHB granules. To exam the correlation between PHB concentration and fluorescence intensity, different concentrations of aqueous PHB suspension were incubated with 2 µg/mL LipidGreen1 for 30 min, and then the fluorescence intensities were measured at 450 nm excitation and 510 nm emission wavelengths, respectively (**a**). On the other hands, intracellular PHB contents were measured by collecting PHB-producing cells every 2 or 3 h during cultivation. The fluorescence intensities were measured with 2.0 µg/ml of LipidGreen1 (**b**) and Nile red (**c**). The PHB contents of the cells at different times were analyzed by GC. The *solid line* indicates the regression line.

The feasibility of LipidGreen1 staining for the quantitative measurement of PHB contents was examined using the aqueous PHB suspension and the PHB-producing cells. The fluorescence intensity of PHB suspension ranging from 0.15 to 5 g/L increased linearly with a high correlation coefficient (R^2^ = 0.96) (Fig. 4a). The cell suspensions prepared from a different amount of PHB-producing *E. coli* cells were analyzed both by LipidGreen1 staining and by GC-based quantification of the purified cellular PHB. There was a good agreement (R^2^ = 0.96) between the fluorescence intensities and the amount of PHB measured by GC (Fig. 4b). The fluorescence intensity by Nile red staining for the purified cellular PHB had a lower relationship (R^2^ = 0.76) to the GC-based amount of PHB (Fig. 4c).

### High-throughput screening of PHB contents in a *phaC* mutant library

**Fig. 5:**
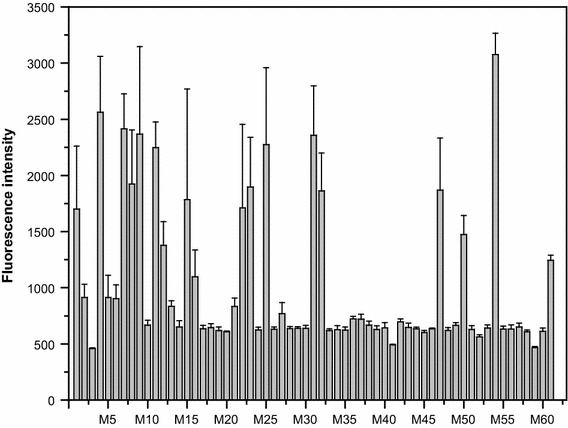
Fluorescence intensities of the mutant library, which consisted of a pool of *phaC* genes with PCR-introduced random mutations. Every single mutant clone was inoculated in a 96-deep well plate and cultivated for 20 h in 800 µl of LB medium with 20 g/L glucose. Fluorescence intensities were monitored after transferring 100 µl of culture solution into a black microplate. The x axis represents the names of the mutants.

The PHA synthase gene (*phaC*) in the PHB biosynthesis gene cluster was randomly mutated by error-prone PCR. A set of *E. coli* clones, each containing a variant of the *phaC* gene, was selected randomly and used to measure the fluorescence intensity of intracellular PHB. When the mutant clones were cultivated in deep-well plates for 20 h and stained with LipidGreen1, the fluorescence intensities of *phaC* variants widely differed from wild type *phaC*, ranging from 500 to 3,000, which obviously was due to the difference in their PHB-synthesizing ability (Fig. 5). When compared to the wild-type *phaC*, about 60% of the mutant clones had lower fluorescence intensities. In contrast, 25% of the *phaC* variants among 60 mutant clones were observed to have higher fluorescence intensities than that of the wild type, and in particular, 2% of the *phaC* variants found had a more than twofold increase in fluorescence intensity. To verify whether the fluorescence intensity of bacterial PHB stained by LipidGreen1 agreed with the intracellular PHB contents, we selected mutant clones that exhibited increased or decreased fluorescence intensities compared to wild type *phaC* and analyzed the PHB contents with GC. PHB accumulations were measured for two clones that showed twofold higher fluorescence intensities (M11 and M54) and for three clones that showed lower fluorescence intensities (M3, M21 and M43) than that of the wild type. Consequently, the mutant clone M54 showing 2.5 times higher fluorescence intensity than wild type *phaC* accumulated about 2.5 times more PHB than that of the wild type *phaC* (Additional file 1: Table S1). The fluorescence intensity of clone M11 producing double amount of PHB contents than wild type *phaC* also showed twice higher values than that of wild type. In contrast, mutant M43 with half the fluorescence intensity of wild type *phaC* had about 10% of the normal PHB accumulation found in wild type, showing a lower detection limit.

## Discussion

It has been often noted that intensities of fluorescence signal by lipophilic dyes were not directly proportional to the amount of lipids (O’Rourke et al. 2009). For example, Nile red poorly stained high fat-containing tissues of *C. elegans* such as germline, eggs, and hypodermis. Furthermore, Nile red and BODIPY were stained the lysosome-related organelles rather than the major *C. elegans* fat storage compartment. In the previous studies on LipidGreen1 and its derivatives, LipidGreen1 was capable of a brighter and less non-specific staining for the detection of neutral lipids and fat deposits compared to the commercially available Nile red and BODIPY^®^ 493/503 (Lee et al. 2011; Chun et al. 2013). Therefore, we attempted to examine a possibility for the more reliable detection of bacterial PHB, one of the promising biodegradable plastics, using the lipophilic fluorescent LipidGreen1.

As shown in Fig. 1, the PHB-producing cells on agar plates containing the fluorescent LipidGreen1 showed the green emission fluorescence under UV irradiation. The result indicated that the LipidGreen1 could enter a bacterial cell probably by diffusion across the cell wall and the inner and outer membranes to the cytoplasm where it subsequently bound to the PHB granules. Furthermore, the LipidGreen1 was not harmful for the bacterial growth on agar plates (Fig. 1) and in liquid medium (Additional file 1: Fig. S3), and thus could be used from the beginning of the bacterial culture. The fluorescent Nile red could be also used to detect PHB in growing bacterial cells by directly including the DMSO-based dye solution in the culture medium (Spiekermann et al. 1999).

The major limitation of Nile red is that the fluorescence intensity gradually diminishes after adding the dye. A significant decrease in fluorescence intensity over 10 min was observed when 0.5% of soybean oil distributed in 0.3% Tween 80 solution was stained with 40 µl of 250 µg/ml Nile red (Montalbo-Lomboy et al. 2014). In addition, the maximum fluorescence intensity of oleaginous yeasts was reached between 1 and 5 min after adding Nile red and slowly faded after 5 min (Kimura et al. 2004). In this study, the cell suspension incubated with 0.8 µg/mL Nile red showed high potency of photo-quenching (Additional file 1: Fig. S2). The fluorescence intensities decreased after 1 h-incubation in cell resuspension even though the fluorescence intensities were almost absent after 19 h. The reason why fluorescence intensity of Nile red steadily decreased during incubation with oil or polyester-accumulating cells was due to its hydrophobic property, which maintained its fluorescence only in nonpolar solvents (Greenspan and Fowler 1985). However, in the case of LipidGreen1, the fluorescence intensities were steadily maintained with only slight differences between the time points at 0.5 and 2 h (Fig. 2). Calculated hydrophobicity (cLogP values) result using Chemdraw program agreed with above data, which hydrophobicity of Nile red (cLogP = 4.6) was higher than that of LipidGreen1 (cLogP = 4.1). The lower hydrophobicity of LipidGreen1 was supposed to contribute maintaining a good fluorescence intensity in aqueous solution by distributed the dye in aqueous solution without loss of its fluorescence intensity. Therefore, LipidGreen1 is a good candidate for a fluorescent probe to monitor PHB accumulation in situ.

Due to the unique and asymmetric lipid composition of bacteria, the outer membrane is fairly impermeable to hydrophobic compounds and moderately so to hydrophilic compounds. Thus, lipophilic dyes such as Nile red and FITC have limited membrane permeability (Herrera et al. 2002). In this study, the fluorescence intensity of the membrane disrupted cells stained with LipidGreen1 was 1.5 times higher than that of intact cells, suggesting insufficient accessibility of the dye to the intracellular PHB (Fig. 3). Previously, the addition of salts or solvents enabling adequate access for the fluorescent molecules to the intracellular PHB such as sucrose and DMSO was suggested to improve the fluorescence intensity and sensitivity (Tyo et al. 2006; Lee et al. 2013; Chen et al. 2009). Nevertheless, the increment of fluorescence intensities was proportional with the PHB concentration to the GC measurement, indicating that the quantitative measurement of intracellular PHB is possible without permeabilization of LipidGreen1 (Fig. 4b). On the contrary, Nile red showed lower correlation coefficient value and specificity to PHB than LipidGreen1 in this experiment (Fig. 4c). We supposed that the exclusion of internalized Nile red in *E. coli* cells lowered the sensitivity and specificity to PHB. Therefore, in term of necessity for internalization, LipidGreen1 had an advantage over Nile red because LipidGreen1 could omit the membrane-permeability procedures for quantitative intracellular PHB measurement.

The screening of mutant library of PHB polymerase in *C. necator* resulted in no clones with a distinctly higher PHB accumulation compared to wild type (Taguchi et al. 2001). PHB accumulation in all the mutant clones was lower than that of the wild type clone, suggesting that the wild-type PHB polymerase is highly optimized for PHB accumulation defined as the ‘fitness landscape model’ (Taguchi et al. 2001). In the same manner, about 200,000 mutants of PHA synthase gene from *Aeromonas punctata* were screened and only five mutants with enhanced fluorescence were isolated (Amara et al. 2002). In this study, most of the mutant clones had much lower or similar fluorescence intensities compared to the wild type consistent with ‘fitness landscape model’. Despite of that, 2% out of total mutant clones showed the distinctively improved fluorescence intensities (Fig. 5). The increased fluorescence intensities of mutant clones agreed with the increment of PHB amount measured by GC. Thus, high-throughput screening of PHB synthase mutant library was available using LipidGreen1 through 96-well microtiter plate-based assay. In addition the PHB amount can be measured with a preparation time of less than 10 min and only a small sample volume (100–200 µL).

In conclusion, quantitative measurement of intracellular PHB with a new fluorescent dye LipidGreen1 was confirmed that PHB accumulation can be visualized in viable colonies by LipidGreen1, providing tools to distinguish between PHB-producing and nonproducing cells. Moreover, LipidGreen1 was highly effective in quantifying PHB by fluorescence measurement because of its prolonged sustainability providing better accuracy and sensitivity than Nile red. A bacterial mutant with enhanced PHB production can be distinguished from randomly mixed samples such as random mutagenesis by error-prone PCR in a high-throughput manner using LipidGreen1. LipidGreen1 could be developed into a commercial kit that rapidly determines the PHB contents, achieving real time monitoring for mass production.

## Additional file

Additional file 1:
**Fig. S1.** Chemical structures of LipidGreen1 and Nile red. **Fig. S2.** The fluorescence intensities of cell suspensions incubated with different concentrations of Nile red. The cell suspension was prepared by resuspending PHB-producing cells into PBS buffer to make OD_600_ 2.0. Two microliter of Nile red solution (0.2, 0.4, 0.6, and 0.8 μg/mL) was added to 1 mL of cell suspension. After further incubation for 0.5, 1, and 19 h in dark place, the fluorescence intensities were measured soon after isolation of the 200 μL of incubated solution. **Fig. S3.** The optical density of cell cultures of *E.coli* XL1-Blue harboring pReCAB under the different concentration of LipidGreen1(A) and Nile red (B). Single colony of PHB-producing cell was inoculated into 2 mL of LB broth and incubated for 20 h at 37°C. The culture was transferred to 100 mL of LB medium containing LipidGreen1 at the final concentrations of 0, 0.8, and 2 μg/mL and further incubated at 37°C with shaking (200 rpm). One milliliter of the culture was isolated every 2 or 3 h to measure the optical density at 600 nm. **Table S1.** PHB accumulation in mutant clones^a^. ^a^The clones showing relatively high and low fluorescence intensities than the wild type were selected to determine PHB contents. PHB contents were measured by GC analysis.
