# Supplementary material for: A lipophilic fluorescent LipidGreen1-based quantification method for high-throughput screening analysis of intracellular poly-3-hydroxybutyrate
Source: AMB Express. 2015 Aug 9;5:48. doi: 10.1186/s13568-015-0131-6 (PMC4529427; doi:10.1186/s13568-015-0131-6)
Supplement: Additional file 1: — Fig. S1. Chemical structures of LipidGreen1 and Nile red. Fig. S2. The fluorescence intensities of cell suspensions incubated with different concentrations of Nile red. The cell suspension was prepared by resuspending PHB-producing cells into PBS buffer to make OD600 2.0. Two microliter of Nile red solution (0.2, 0.4, 0.6, and 0.8 μg/mL) was added to 1 mL of cell suspension. After further incubation for 0.5, 1, and 19 h in dark place, the fluorescence intensities were measured soon after isolation of the 200 μL of incubated solution. Fig. S3. The optical density of cell cultures of E.coli XL1-Blue harboring pReCAB under the different concentration of LipidGreen1(A) and Nile red (B). Single colony of PHB-producing cell was inoculated into 2 mL of LB broth and incubated for 20 h at 37°C. The culture was transferred to 100 mL of LB medium containing LipidGreen1 at the final concentrations of 0, 0.8, and 2 μg/mL and further incubated at 37°C with shaking (200 rpm). One milliliter of the culture was isolated every 2 or 3 h to measure the optical density at 600 nm. Table S1. PHB accumulation in mutant clonesa. aThe clones showing relatively high and low fluorescence intensities than the wild type were selected to determine PHB contents. PHB contents were measured by GC analysis. [file 13568_2015_131_MOESM1_ESM.pdf]

Supporting information

A lipophilic fluorescent LipidGreen-based quantification method for high-throughput screening analysis of intracellular poly-3-hydroxybutyrate

Ji-Eun Choi[1], Hye Young Na[1,2], Taek Ho Yang[3], Sung-Keun Rhee[2] and Jae Kwang Song[1]*

[1]Research Center for Bio-based Chemistry, Korea Research Institute of Chemical Technology (KRICT), 141 Gajeong-ro, Yuseong-gu, Daejeon 305-600, Republic of Korea

[2]Department of Microbiology, Chungbuk National University, Chungdae-ro 1, Seowon-gu, Cheongju, Chungbuk 362-763, Republic of Korea

[3]GenoFocus, 65 Techno 1-ro, Yusung-gu, Daejeon 305-509, Republic of Korea

* Correspondence: ajee@krict.re.kr (J.K. Song)

[1]Research Center for Bio-based Chemistry, Korea Research Institute of Chemical Technology (KRICT), 141 Gajeong-ro, Yuseong-gu, Daejeon 305-600, Republic of Korea

This supporting information has 6 pages and includes four figures and one table namely Figure S1, S2, S3, and Table S1.

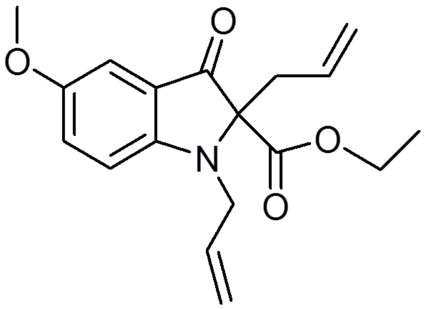

LipidGreen1

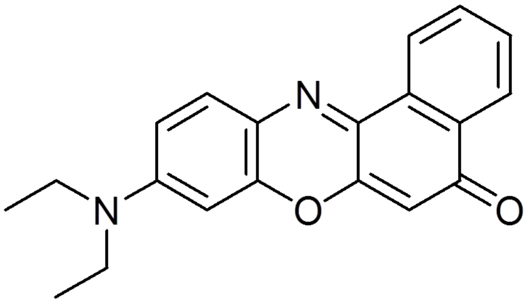

Nile red

Fig. S1 Chemical structures of LipidGreen1 and Nile red.

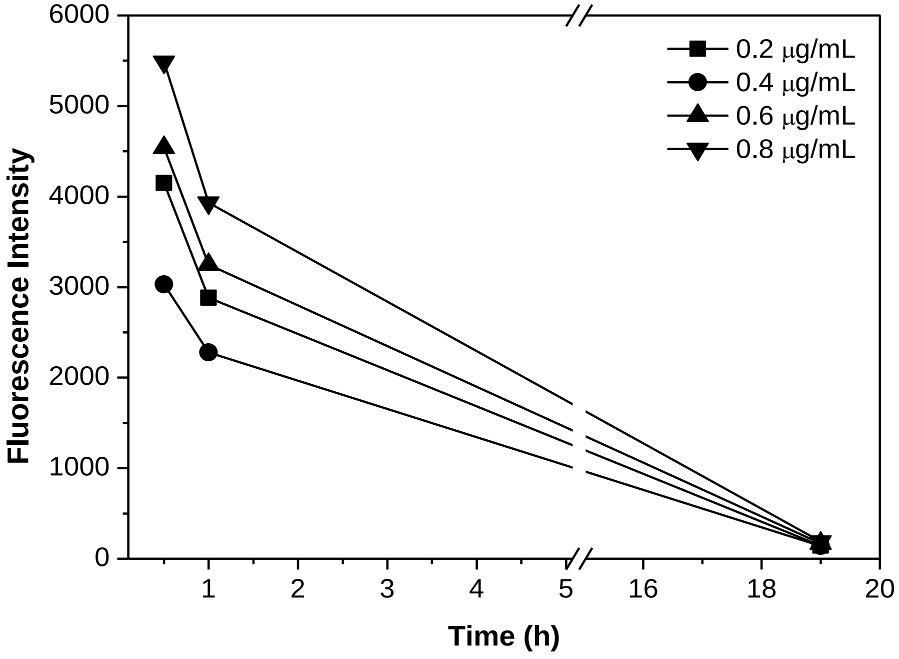

Fig. S2 The fluorescence intensities of cell suspensions incubated with different concentrations of Nile red. The cell suspension was prepared by resuspending PHB-producing cells into PBS buffer to make OD_{600} 2.0. Two microliter of Nile red solution (0.2, 0.4, 0.6, and 0.8 μg/mL) was added to 1 mL of cell suspension. After further incubation for 0.5, 1, and 19 h in dark place, the fluorescence intensities were measured soon after isolation of the 200 μL of incubated solution.

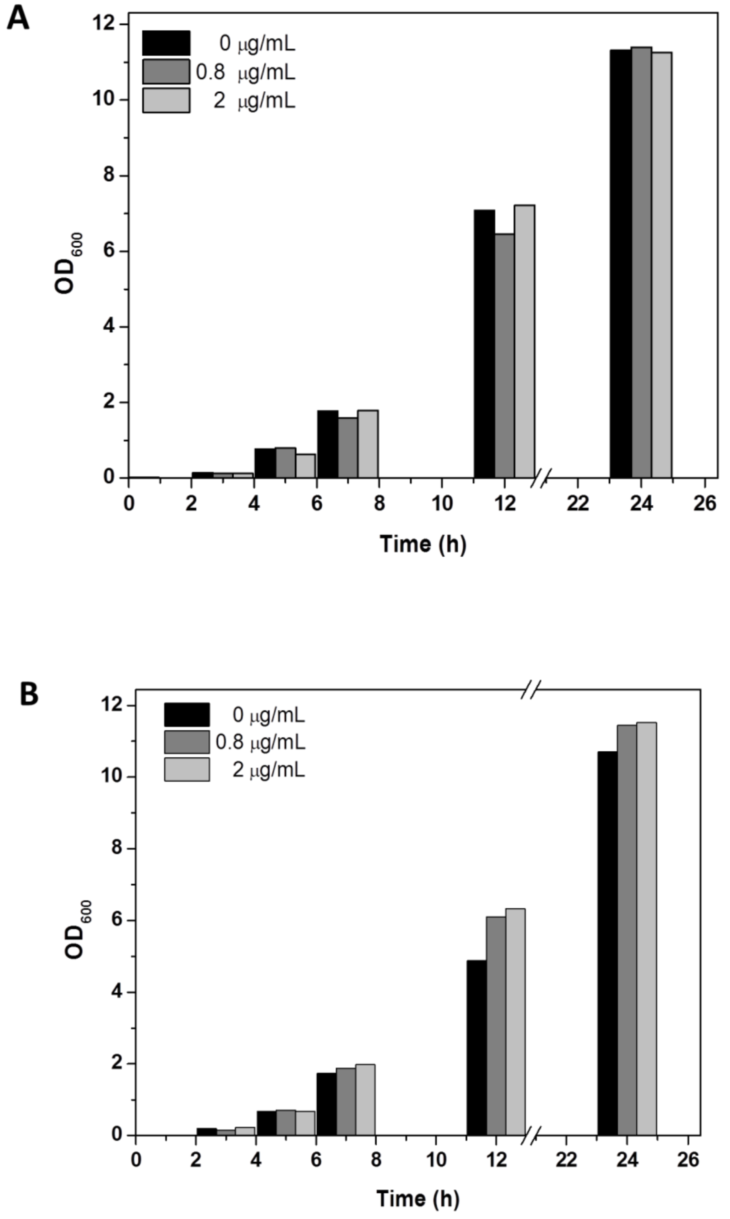

Fig. S3 The optical density of cell cultures of *E.coli* XL1-Blue harboring pReCAB under the different concentration of LipidGreen1(A) and Nile red (B). Single colony of PHB-producing cell was inoculated into 2 mL of LB broth and incubated for 20 h at 37°C. The culture was transferred to 100 mL of LB medium containing LipidGreen1 at the final concentrations of 0,

0.8, and 2 μg/mL and further incubated at 37°C with shaking (200 rpm). One milliliter of the culture was isolated every 2 or 3 h to measure the optical density at 600nm.

Table S1 PHB accumulation in mutant clones[a].

Mutant	PHB contents (%)
M3	0.27
M11	36.33
M21	4.98
M43	2.87
M50	17.75
M54	48.14
Wild-type	17.85

[a] The clones showing relatively high and low fluorescence intensities than the wild type were selected to determine PHB contents. PHB contents were measured by GC analysis.
